# The Use of a Non-Invasive Biomarker for Colorectal Cancer Screening: A Comparative Cost-Effectiveness Modeling Study

**DOI:** 10.3390/cancers15030633

**Published:** 2023-01-19

**Authors:** Martin C. S. Wong, Junjie Huang, Yuet-Yan Wong, Samantha Ko, Victor C. W. Chan, Siew C. Ng, Francis K. L. Chan

**Affiliations:** 1 JC School of Public Health and Primary Care, Faculty of Medicine, The Chinese University of Hong Kong, Hong Kong, China; 2 Centre for Health Education and Health Promotion, Faculty of Medicine, The Chinese University of Hong Kong, Hong Kong, China; 3 Centre for Gut Microbiota Research, Faculty of Medicine, The Chinese University of Hong Kong, Hong Kong, China; 4 The Department of Medicine and Therapeutics, The Chinese University of Hong Kong, Hong Kong, China

**Keywords:** colorectal cancer, screening, fecal immunochemical tests, non-invasive biomarker, cost-effectiveness

## Abstract

**Simple Summary:**

There is a scarcity of economic analysis on the use of non-invasive biomarkers to inform policy-making with respect to their application in population-based colorectal cancer (CRC) screening programs. The objective of this study is to conduct a comparative cost-effectiveness analysis on using M3CRC (m3 + Fn + Ch + Bc + FIT) as a primary test for population-based screening, when compared with FIT and colonoscopy. The study can provide economic analysis regarding the use of non-invasive biomarkers in population-based CRC screening programs.

**Abstract:**

This study aimed to examine the cost-effectiveness of fecal biomarker M3 panel compared to fecal immunochemical test (FIT) and colonoscopy in an Asian population. In a hypothetical population of 100,000 persons aged 50 years who received FIT yearly, M3 biomarker yearly, or colonoscopy every 10 years until the age of 75 years. Participants with positive FOBT or a result of “high risk” identified using the M3 biomarker are offered colonoscopy. We assumed surveillance colonoscopy is repeated every 3 years, and examined the treatment cost. A comparison of various outcome measures was conducted using Markov modelling. The incremental cost-effectiveness ratio (ICER) of FIT, M3 biomarker, and colonoscopy was USD108,176, USD133,485 and USD159,596, respectively. Comparing with FIT, the use of M3 biomarker could lead to significantly smaller total loss of cancer-related life-years (2783 vs. 5279); a higher number of CRC cases prevented (1622 vs. 146), a higher proportion of CRC cases prevented (50.2% vs. 4.5%), more life-years saved (2852 vs. 339), and cheaper total costs per life-year saved (USD212,553 vs. 773,894). The total costs per life-year saved is more affordable than that achieved by colonoscopy as a primary screening tool (USD212,553 vs. USD236,909). The findings show that M3 biomarkers may be more cost-effective than colonoscopy.

## 1. Introduction

Colorectal cancer (CRC) constitutes a substantial proportion of disease burden globally, and its incidence continues to out-rank other cancers in developed countries [1]. The overall prevalence rates of adenoma (23.9%; 95% CI, 22.2–25.8%), advanced adenoma (4.6%; 95% CI, 3.8–5.5%), and CRC (0.4%, 95% CI, 0.3–0.5%) remain high worldwide [2]. Over the past decade, there has been an increasing trend of CRC incidence in Asian countries, which necessitates more intensive preventive initiatives [3]. CRC screening by fecal immunochemical tests (FITs) and colonoscopy has reduced CRC-related mortality by 33% and 68%, respectively [4,5,6]. Although colonoscopy is the most sensitive test for detecting CRC and premalignant lesions, its high cost and invasive nature render its first-line use challenging in most Asian countries. Conversely, screening programs based on FITs have suffered from a low participation rate (global average: 54%, 95% CI 49.3% to 58.7%), attributable partly to the inability of the FITs to detect adenomatous polyps and their low sensitivities for CRC (0.79, 95% CI 0.69 to 0.86) [7,8,9].

The adoption of non-invasive biomarker may potentially increase participation rate of CRC screening due to its high acceptance [10]. Additionally, an accurate non-invasive biomarker test could be an attractive alternative for asymptomatic, average-risk participants who are keen to detect colorectal adenoma and CRC at earlier stages—since endoscopic polypectomy of colorectal adenomas could significantly decrease the risk of CRC development [11]. A recent novel fecal bacterial biomarker named M3CRC was identified as a non-invasive screening test for both lesions. The combination of Lachnoclostridium species marker m3, Fusobacterium nucleatum (Fn), Clostridium hathewayi (Ch), Bacteroides clarus (Bc), and a FIT can achieve good diagnostic performance for CRC (sensitivity: 93.8%; specificity: 81.2%) and adenoma (sensitivity: 47.1%; specificity: 81.2%) [12]. Nevertheless, there is a scarcity of economic analysis on use of non-invasive biomarkers to inform policy-making with respect to their application in population-based CRC screening programs, as previous cost-effectiveness analyses (CEA) mainly focused on biomarkers that could detect CRC only [13,14,15,16,17,18,19,20,21].

We have previously conducted economic evaluations of CRC screening based on FIT in various population subgroups [22,23,24,25], and built a statistical model that allows use of different parameters of screening tests to compute different cost-effectiveness outcomes. The objective of this research is to conduct a comparative cost-effectiveness study based on modelled data and assumptions on using M3 (m3 + Fn + Ch + Bc + FIT) as a primary test for population-based screening, when compared with FIT and colonoscopy.

## 2. Materials and Methods

### 2.1. Decision Model Framework

**Figure 1:**
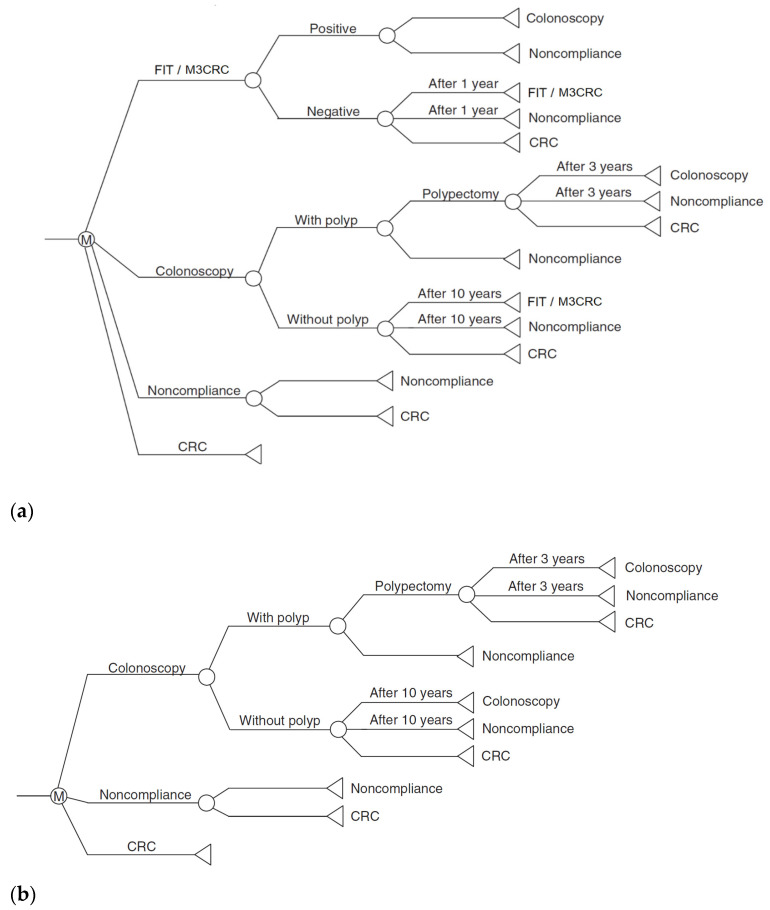
Markov process on strategies using FIT, M3CRC and colonoscopy as primary screening test. They are listed as: (**a**) FIT/M3CRC; (**b**) Colonoscopy. CRC: colorectal cancer; FIT: fecal immunochemical tests. The figure described transitions of screening participants to various stages, and the model included annual mortality. Subjects were followed up from 50 to 75 years.

A decision analysis algorithm based on a Markov model was used for data analysis (Figure 1). We supposed a population of 100,000 individuals who aged 50 years and had no past history or symptoms of CRC. Three distinct screening strategies included M3CRC, FIT, and colonoscopy following up until the age of 75 years. The strategies were given to the entire population, and were compared to no screening with reference to a previous CEA [26].

Strategy 1: FIT as primary screening test. Each subject will receive one specimen FIT with the test repeated yearly among those with negative FIT. Those with positive FIT are offered colonoscopy workup. Subjects with normal colonoscopy will resume FIT after 10 years, whilst those with polyps removed will have a surveillance colonoscopy arranged every 3 years until there are no more polyps. 

Strategy 2: M3CRC as primary screening test. Each screening participant will be offered one M3CRC on a yearly basis amongst those with average or moderate risk reports. Those with results of “high risk” will be offered colonoscopy workup. Subjects with normal colonoscopy will resume M3CRC after 10 years, whilst those with polyps removed will have a surveillance colonoscopy arranged every 3 years until there are no more polyps. 

Strategy 3: Colonoscopy as primary screening test. Every screening participant received one direct colonoscopy. Those with normal results will receive a repeat colonoscopy after 10 years, whilst those with polyps removed during colonoscopy will be offered a surveillance colonoscopy every 3 years until no more polyps are detected, by which a repeat colonoscopy will be scheduled 10 years later.

### 2.2. Clinical Transition Probabilities

The transition probabilities constructed into the models were derived from published literature in Asian countries (Table 1). First, the sensitivity and specificity of FIT in detecting CRC was set at 73.0% and 91.9% whilst the corresponding figures for M3CRC in detecting a composite measure of non-advanced adenoma, advanced adenoma and CRC was 66.5% and 83.8% (personal communication, Dr. Jessie Liang) [16,27]. Second, the compliance rates of FIT, M3CRC and colonoscopy were estimated based on figures from a large Asian population [12,16,22]. The compliance rates of CRC screening tests are different among Asian countries. The compliance rates of FIT in this study referred to a previous study on the cost-effectiveness of screening with the MT-sDNA test, FIT or colonoscopy. The compliance rate was 38–60% with 73% sensitivity, 96% specificity, and USD19-unit cost [16]. In this study, the compliance rate of 60% was used. The compliance rate of M3CRC was the compliance with valid test results after issuance of the screening test 99% based on a Markov model [12]. The compliance rates on the screening tests were assumed in the base-case model with a range tested from 10% to 100% by using a sensitivity analysis. It was assumed that the compliance rate to colonoscopy was 100% after a positive result on M3 or FIT was received [28]. The sum of true- and false-positive cases was used to calculate the rate of positive results on M3CRC or FIT. Third, the rate of polypectomy of FIT, that of M3CRC, polypectomy bleeding rate, and polypectomy perforation rate were 73.0%, 83.9%, 0.98%, and 0.08% [29,30]. The mortality due to perforation was taken as 0.0029%, with reference to a systematic review [30]. Screening intervals were selected based on the assumptions from a previous CEA [26].

### 2.3. Studies on Diagnostic Accuracy for M3

The sensitivity and specificity of M3 have been examined in a previous study that included 1012 subjects from two independent Asian groups [12]. The M3 biomarker panel consists of a Lachnoclostridium sp. Marker m3, Fusobacterium nucleatum (Fn), Clostridium hathewayi (Ch) and Bacteroides clarus (Bc) for non-invasive diagnosis of colorectal neoplasia. The test involves collection of fecal materials at home which are put into collection tubes, which is similar to that of FIT. The test kits with the saved fecal materials are then sent to an accredited laboratory for metagenomics sequencing and quantitative Polymerase Chain Reaction (qPCR). The details of the collection procedure were published elsewhere [12]. It could achieve a good diagnostic performance for CRC (sensitivity: 93.8%; specificity: 81.2%) and adenoma (sensitivity: 47.1%; specificity: 81.2%). In a subgroup tested with fecal immunochemical test (FIT) for non-invasive diagnosis of colorectal adenoma, M3 had better performance than FIT in detecting adenoma. Sensitivities for non-advanced and advanced adenomas by M3 were 44.2% and 50.8% at a specificity of 79.6%, while that of FIT were 0% and 16.1% at specificity of 98.5% among 642 subjects. Similar to M3, FIT is also a non-invasive stool test which was most widely used. However, only 16.1% of advanced adenoma and none of the nonadvanced adenoma could be detected by FIT. On the contrary, there was no significant difference in the detection rate between advanced and non-advanced adenomas by M3 with sensitivities of 50.8% and 44.2%, respectively, at a sensitivity of 48.3% in detecting adenoma by M3. M3 could perform better in detecting non-advanced adenoma when compared with all other available stool-based tests. The detection rate of advanced adenoma by M3 increased from 50.8% to 56.8% when combined with FIT.

According to the yearly age-specific incidence rates of CRC in Hong Kong, CRC can be developed in the population if screening was not taken [31]. By contrast, earlier stages detection of CRC can improve the overall survival rate. In a network meta-analysis, the incidence rate of CRC can be reduced by 21% [32]. After yearly CRC screening by colonoscopy or M3CRC, cases should be reduced to be 51.5% and 54% from a territory-wide CRC screening program in Hong Kong and a previous CEA performed by our team [22].

The Hong Kong Cancer Registry has announced the annual mortality rates of CRC diagnosed at different stages, which have been used as reference in this study [31,33]. First, patient in stage I and II CRC receive only surgery with an intent to cure (recurrence-free for at least three to five years). Second, patient in stage III disease receive adjuvant chemotherapy after surgery. It is expected that 70% of patients would be cured while 30% of the patients would develop a recurrent disease. Palliative colorectal surgery is given to patient in stage IV disease, following by palliative chemotherapy. Half of these patients develop liver metastasis and need further surgery [26]. There are five common and available regimens, which are FOLFIRI, FOLFOX, Cetuximab, 5FUFA, Avastin. In a metastatic and adjuvant setting, 5FUFA is viewed as the first-line agent. FOLFOX + bevacizumab (Avastin; Roche/Genentech Inc., San Francisco, CA, USA) for 10 months and FOLFOX for 6 months were assumed to be used according to the base-case model in this study. This is because the two agents are currently the most effective chemotherapeutic agents for patients in stage III and IV diseases in the same setting [34,35,36,37]. Follow-up surveillance and managing possible cancer recurrence would be ignored when patients are ‘cured’ of CRC in this model.

### 2.4. Cost Estimates

US dollars are used as the unit of the cost. The decision model has been used and it included direct costs of screening tests, cost of investigation and staging of CRC inclusive of Computed Tomography (CT) and Positron Emission Tomography (PET) scans, cost of cancer treatment inclusive of surgery and chemotherapy, along with hospitalization costs (Table 2). Diagnosis and pre-operative assessment included health services of colonoscopy with/without biopsy, histopathological examination, carcinoembryonic antigen level, CT contrast scan for abdomen and pelvis, ultrasound of abdomen, Magnetic Resonance Imaging (MRI) contrast scan for pelvis, PET scan, and outpatient specialist clinic. Follow-up included health services of carcinoembryonic antigen level, ultrasound of abdomen, and outpatient specialist clinic. The possibility of hospitalization due to complications (perforation or bleeding) after polypectomy and/or endoscopy were included in the cost. The hospitalization included labor costs for disposable instruments and daily hospital care while the costs of surgical procedures and consultation, and the costs of CT and PET scan were separately calculated. It was assumed that the average period of hospitalization for patients who had with surgery was nine days [34]. About the exclusion criteria, indirect costs were excluded in the simplicity analysis, including productivity lost due to absence of work and transportation costs to hospital. The costs of blood transfusions and repeat visits were also excluded. About future costs resulting from screening or care of CRC, and the future life-years saved by screening are discounted yearly at 3% [38].

### 2.5. Sensitivity Analysis

The health cost was different among Asian countries, therefore, robustness across different intervals of key parameters were evaluated by ICER sensitivity analyses. It included sensitivity of M3CRC, compliance rates of screening tests, and cost of colonoscopy. One-way sensitivity analyses on ICER were conducted to compare between different screening strategies with the range of model variables. Compliance rates on initial, repeated and follow-up screening are equal. The threshold values for the change of conclusion were shown when the results are not robust. Excel spreadsheets were used to simulate all calculations.

### 2.6. Cost-Effectiveness Analysis

Life-years can be saved through CRC prevention and improvement on survival rate due to earlier cancer diagnosis. Cost-effectiveness is measured in terms of these life-years being saved. By contrast, life-years loss is measured by using the Hong Kong standard life table. The life-years saved and the cancer-related deaths would be examined with and without Markov model. The main outcome would be the incremental cost-effectiveness ratio (ICER) between different screening strategies, a measurement to quantify the amount of additional cost required for a life-year saved.

## 3. Results

The results of four actions: FIT, M3CRC, colonoscopy, and no screening, were represented in Table 3. A yearly discount rate of 3% was included in the number of life-years saved and lost, the cases of CRC prevented and the costs associated with the strategies. If no screening was given, there will be 3233 cases of CRC and 5635 cancer-related life-years losing among 50-year-old subjects. When FIT, M3CRC and colonoscopy were performed, the proportion of CRC case prevented were 4.5%, 50.2%, and 51.3%, respectively. Screening with colonoscopy could save more life-years and reduce more death at a higher cost while the cost of treatment for advanced CRC (stage III and IV) was higher. Reducing cancer-related death and treatment expenditures were the results of earlier detection of cancer by screening.

**Figure 2:**
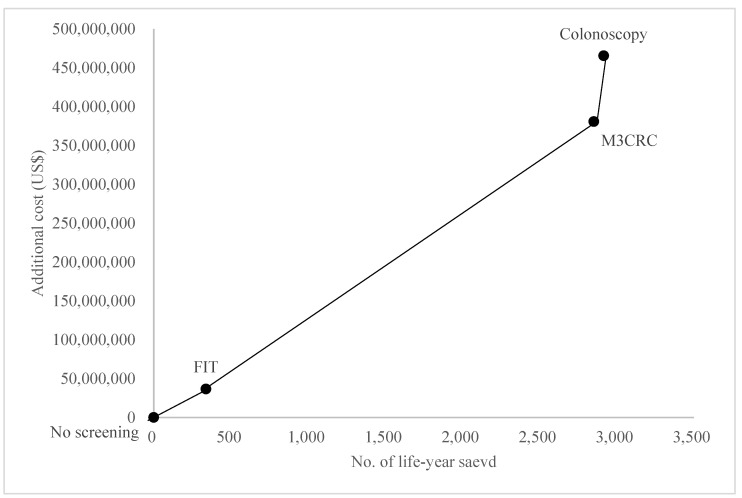
Cost-effectiveness analysis for the screening tests.

**Figure 3:**
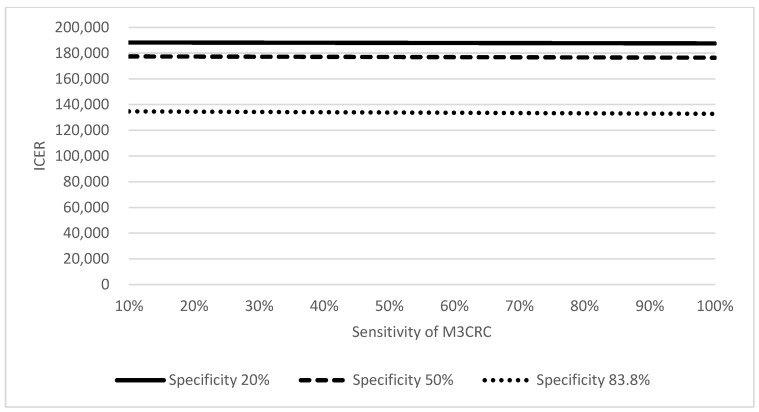
One-way sensitivity analysis on the compliance rate of screening tests.

The total costs for the management of CRC increase from no screening (USD226 million) to FIT (USD262 million), to M3CRC (USD606 million), and to colonoscopy (USD691 million). Comparing to no-screening, ICER of FOBT, FS and colonoscopy are USD108,176, USD133,485 and USD159,586, respectively (Table 3, Figure 2), which meant that FIT was most cost-effective among different strategies to prevent and manage CRC. The compliance rates of M3CRC, FIT, and colonoscopy were 60%, 99%, and 98.9%, respectively, in the base-case model. A range of compliance rates was tested by one-way sensitivity analysis (Figure 3). ICER was increased with a reduced compliance rate, which indicated that extra costs were required to save one life-year. However, when compared to colonoscopy and M3CRC, FIT still had the lowest ICER.

**Figure 4:**
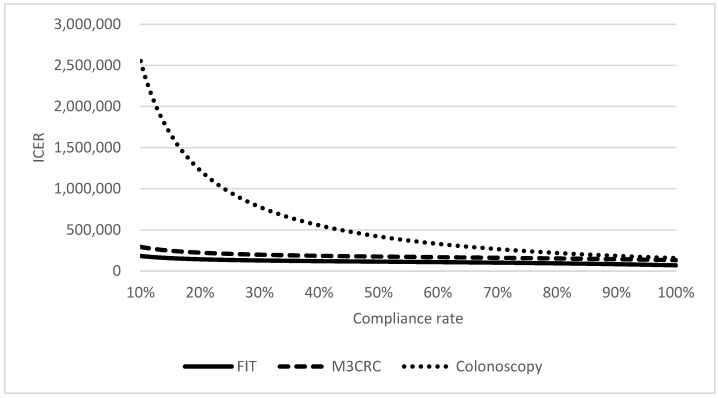
Two-way sensitivity analysis on the sensitivity and specificity of M3CRC.

Cost-effectiveness of M3CRC was determined by its performance. In the base-case model, the sensitivity and specificity of M3CRC were 66.5% and 83.8%, respectively [12]. ICER of M3CRC increased while the specificity decreased from 83.8% to 20% (Figure 4). Similarly, ICER of M3CRC increased slightly when the sensitivity reduced. When the specificity reduced to 20–50%, ICER of M3CRC exceeded that of colonoscopy indicating that M3CRC is less cost-effective at lower specificity.

**Figure 5:**
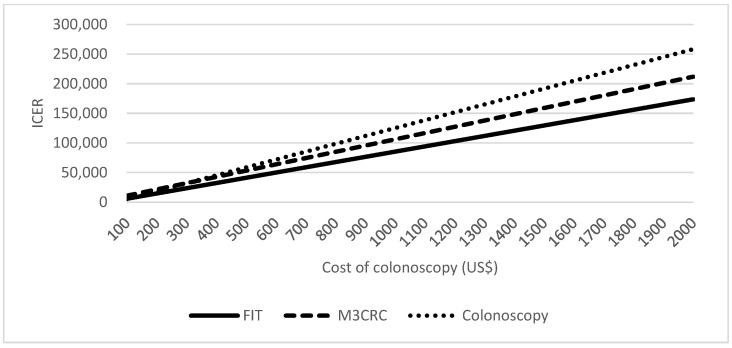
One-way sensitivity analysis on the cost of colonoscopy.

M3CRC was more cost-effective than direct colonoscopy as a primary screening tool. This was not affected much by the cost of colonoscopy. At the range of USD305 to USD2000 per colonoscopy, ICER of M3CRC remains lower than that of colonoscopy (Figure 5).

ICERs could be affected by a higher treatment cost for CRC. When the treatment cost for stage IV CRC increased to USD139,591, ICERs of M3CRC, FIT, and colonoscopy were USD70,228, USD49,034, and USD96,334, respectively. Although the difference between ICERs of the screening strategies were closer, FIT as the most cost-effective strategy for CRC screening was not affected.

ICERs increased with the discount rate. When the discount rate increased to 6%, ICERs of M3CRC and colonoscopy were USD122,052 and USD154,577, respectively. The difference between ICERs of the screening strategies has been enlarged, and M3CRC was still more cost-effective than colonoscopy.

## 4. Discussion

### 4.1. Major Findings

This study compared FIT, M3 biomarker, and colonoscopy screening methods. First, this study showed that FIT was the more cost-effective as it had the lowest ICER of USD108,176 as compared to M3 and colonoscopy, which were USD133,485 and USD159,586, respectively. However, FIT had the lowest compliance rates across the three screening methods. By contrast, M3 had higher compliance rates (99%) compared with FIT (60%). Second, M3 also had higher compliance rates when compared with colonoscopy (98.9%) with higher performance including sensitivity and specificity (66.5%, 83.8%). More life-years could be saved by M3 at lower total costs (USD212,553) than colonoscopy (USD236,909). Our study found that M3 was more cost-effective than colonoscopy. It also demonstrated that M3 could prevent more CRC cases than FIT although FIT was more cost-effective than M3.

### 4.2. Limitations of FIT

Many studies have examined cost-effectiveness of FIT as the major advantage of this screening method for population screening. A systematic review compared cost-effectiveness between FIT and colonoscopy in CRC screening by examining six randomized controlled trials and 17 cost-effectiveness studies around the world [39,40]. Most studies have found that annual or biennial FIT had higher participation rate and was more cost effective than colonoscopy, for example, ICER was ≤USD25,000 per quality Adjusted Life-Years Gained (QALYG) when compared with colonoscopy every ten years. Our result also showed that FIT was the most cost-effective strategy compared with M3CRC and colonoscopy. Another study also compared the cost-effectiveness of annual FIT with colonoscopy, and other screening strategies including computed tomography (CT), mtSDNA, PillCam, and mSEPT9. When simulating screening from age 50 years to 75 years with perfect adherence and a willingness-to-pay threshold of USD100,000 per QALYG, annual FIT screening was cost-saving compared to no screening and had 26% lower total costs and 63% lower test burden in terms of number of diagnostic colonoscopies when compared to colonoscopy screening. However, the study also implied that regardless of screening strategies, the number of CRC cases and deaths could be reduced from 108 to 37–59 CRC cases, and from 45 to 8–15 deaths. CRC screening could reduce CRC cases and prevent more deaths than no screening, which was not the only advantage of FIT screening method [41]. 

Although FIT was a more cost-effective strategy amongst different screening methods, FIT had a significant limitation in the detection of early-stage CRC. It has been found that FIT was not sensitive for detecting adenoma [42]. A previous study that examined diagnostic accuracy of FIT and M3 has found that only 16.1% advanced adenoma and none of nonadvanced adenoma could be detected by FIT. It had less ideal performance when compared with M3 in detecting adenoma as sensitivities for non-advanced and advanced adenomas by M3 were 44.2% and 50.8%, respectively, at a specificity of 79.6% [12]. Despite its cost-effectiveness, its detection rate of CRC, advanced adenoma, and any adenoma was lower than that of colonoscopy [39]. When compliance rate is reduced, it means that extra costs are required to save a life-year, therefore, ICER would increase accordingly. In our study, it has also been found that FIT had the lowest compliance rate among different screening methods (60%), while M3CRC had the highest compliance rate (99%), following by colonoscopy (98.9%). The sensitivity of FIT would vary between 6–56% for advanced adenoma. Some studies with over 8000 cohort sizes have all demonstrated that the sensitivity of FIT was lower than 28% [43,44,45].

Moreover, FIT had a lower specificity that might lead to false-positive results [42]. A community-based study in Canada found that the predictive ability of FIT screening for colonic adenoma was suboptimal (AUC: 0.60, 95% confidence interval: 0.54–0.65). The sensitivity for advanced adenoma was only 49.5% and that of specificity was 62.7% by the commonly used cutoff level of 75 ng/mL. It stated that patients who were FIT negative also had colonoscopy [44,45]. Owing to lower sensitivity and specificity, cost-effectiveness of FIT screening might not fully reflect the usage and expectation of screening for colonic adenoma among the screening participants.

### 4.3. Strengths of M3CRC When Compared with FIT and Colonoscopy

M3CRC, a stool-based microbial biomarker, had a higher detection rate than FIT in detecting colorectal neoplasia. In our results, M3CRC could prevent 50.2% of CRCs, which is a slightly lower percentage than colonoscopy (51.3%), but it could detect substantially more CRCs cases than FIT (4.5%). From a study that involved 1012 Asian subjects, metagenomic analysis has identified certain microbial markers to be significantly enriched in adenoma. A significant linear trend of the abundance of microbial markers was found to be increasing from control to adenoma to cancer. More importantly, the study has also demonstrated a high sensitivity (62.1%, 48.3%) and specificity (78.5%, 78.5%) of M3 for CRC and adenoma. In diagnosing colorectal adenoma, it has been argued that m3 had better performance than other bacterial markers including Fusobacterium nucleatum (Fn) and Clostridium hathewayi (Ch) [12]. In our study, M3CRC also had a high sensitivity (66.5%) and specificity (83.8%) in the base-case model. When comparing microbial markers with FIT, another study involving 676 subjects has also examined four microbial markers: Fusobacterium nucleatum, Clostridium hathewayi, a Lachnoclostridium sp. m3, and Bacteroides clarus for detection of advanced adenoma and non-advanced adenomas. In quantitative PCR analysis, the combined score of these four microbial markers has been found to be more sensitive in diagnosing CRC and advanced adenoma than FIT [45,46]. These studies have stated that M3CRC had better validation and performance in diagnosing colorectal adenoma.

M3CRC has high capability in detecting recurrent adenomas. A 10-year polyp surveillance study has found that m3 had a significant increase in follow-up versus baseline samples among patients who were diagnosed with adenoma recurrence. The area under receiver operating characteristic curve (AUROC) for changes of m3, Fn, and Ch were found to be 0.95 (95% CI: 0.84–0.99), with high sensitivity and specificity (81.3%, 55.4%) in detecting recurrent adenoma [47]. The capability of detecting recurrent adenomas have been recognized in the use of M3CRC. Although some studies might find compliance issues on stool-based microbial biomarker test, which was similar to other fecal screening modalities, our study has found a positive result on the compliance rate of M3CRC when compared with FIT and colonoscopy [48]. In our study, the compliance rate of M3CRC was nearly 99%, which was higher than that of colonoscopy (98.9%), followed by FIT who had the lowest compliance rate (60%). Further research should be performed to examine the compliance rate of M3CRC in other population groups.

M3CRC was more cost-effective than direct colonoscopy as a primary screening tool. Our findings showed that the ICER of M3CRC was lower than that of colonoscopy at the range of USD305 to USD2000. At a higher CRC treatment cost at USD139,591, M3CRC had a lower ICER (USD70,228) than colonoscopy (USD96,334) although it was higher than that of FIT (USD49,034). With better diagnostic sensitivity, an expectation of new screening tests based on fecal biomarkers to replace FIT with higher cost-effectiveness has been raised by a recent literature [45]. It has been supported by a national survey stating that uninsured people would choose stool-based tests over colonoscopy for CRC screening (OR: 2.53; 95% CI: 1.22–5.65 and OR: 2.73; 95% CI: 1.13–7.47) [45,49]. Therefore, as M3CRC was more cost-effective than colonoscopy and had higher detection rates than FIT, some studies have recommended the use of novel biomarkers to achieve non-invasive diagnosis of colorectal adenoma [12,45,46,47,48].

A cost-effectiveness study that based on a hypothetical population had many assumptions in using the three screening methods. The clinical evidence of the examined methods, especially the actual adherence rate has not been reflected in our study. Besides, the study did not include the diagnostic accuracy of the screening strategies. There is a need to further examine the compliance rates with the cost-effectiveness estimations, and the diagnostic accuracy of the screening strategies. Additionally, individual differences that might require different screening strategies for patients would affect the adherence rates, while various CRC screening strategies should still be presented to patients by health care professionals.

## 5. Conclusions

This study has compared the cost-effectiveness and sensitivity among three CRC screening methods including FIT, M3CRC, and colonoscopy. Although FIT was the most cost-effective strategy, it had low detective rates and sensitivity, which could lead to false-positive and false-negative results. In contrast, the use of M3 biomarker could lead to significantly smaller total loss of cancer-related life-years; a higher number of CRC cases prevented; a higher proportion of CRC cases prevented; more life-years saved; and cheaper total costs per life-year saved. The total costs per life-year saved is even more affordable than that achieved by colonoscopy as a primary screening tool. These findings show that M3 biomarkers may be more cost-effective than colonoscopy when used as a primary screening test in an Asian population.

## Figures and Tables

**Table 1:** Baseline estimates for the screening strategies.

Rate	Estimate	Reference
Sensitivity of FIT (cutoff value = 20 µg/g) in detecting colorectal cancer	73.0%	[16]
Specificity of FIT (cutoff value = 20 µg/g) in detecting colorectal cancer	91.9%	[27]
Sensitivity of M3CRC in detecting a composite measure of NAA + AA + CRC	66.5%	[12]
Specificity of M3CRC in detecting a composite measure of NAA + AA + CRC	83.8%	[12]
Compliance rate of FIT	60.0%	[16]
Compliance rate of M3CRC	99.0%	[12]
Compliance of Colonoscopy	98.9%	[22]
Compliance rate of Colonoscopy after a positive result	100%	[28]
Rate of polypectomy of FIT	73.0%	[29]
Rate of polypectomy of M3CRC	83.9%	[12]
Polypectomy bleeding rate	0.98%	[30]
Polypectomy perforation rate	0.08%	[30]
Morality due to perforation	0.0029%	[30]
Cancer prevented by FIT	21.0%	[31]
Cancer prevented by Colonoscopy	54.0%	[22]
Cancer prevented by M3CRC	51.5%	[12]
Staging of CRC at diagnosis		
I	11.3%	[32]
II	25.4%	[32]
III	32.4%	[32]
IV	31.0%	[32]
Annual mortality of CRC patients at various stages of disease (1 year)		
I	1.0%	[33]
II	4.5%	[33]
III	8.7%	[33]
IV	43.0%	[33]

CRC: colorectal cancer; FIT: fecal immunochemical tests.

**Table 2:** Estimates for the costs based on different screening strategies and treatment methods.

Cost Item	Baseline Value (USD)	Reference
One kit of FIT	19	[16]
M3CRC	64	Internal source
Colonoscopy	1259	[26,34]
Consultation fee	96	[26,34]
Bleeding	3320	[26,34]
Histopathological examination	142	[26,34]
Perforation	10,790	[26,34]
Treatment for the stage I of CRC	17,071	[26,34]
Diagnosis	6091	[26,34]
Treatment	10,377	[26,34]
Follow-up	603	[26,34]
Treatment for the stage II of CRC	19,755	[26,34]
Diagnosis	6091	[26,34]
Treatment	13,061	[26,34]
Follow-up	603	[26,34]
Treatment for the stage III of CRC	26,883	[26,34]
Diagnosis	6091	[26,34]
Treatment	20,189	[26,34]
Follow-up	603	[26,34]
Treatment for the stage IV of CRC	45,115	[26,34]
Diagnosis	6091	[26,34]
Treatment	38,422	[26,34]
Follow-up	603	[26,34]

CRC: colorectal cancer; FIT: fecal immunochemical tests. More parameters used in the model: The age-specific incidence of CRC for general population without screening was retrieved from the Hong Kong Cancer Registry, which were 55.9, 89.8, 137, 200, 253.2 and 330.5 per 100,000 for quinquennial age range from 50 to 75 years old [Hong Kong Cancer Registry Hong Kong Cancer Statistics Available online: https://www3.ha.org.hk/cancereg/allagesresult.asp (accessed on 29 March 2022)]. The mortality rate for each age is based on reference [26].

**Table 3:** Result of 100,000 average-risk individuals aged 50–75 years with FIT, M3CRC, colonoscopy, and no screening for colorectal cancer.

Screening Method	No Screening	FIT	M3CRC	Colonoscopy
Total number CRC cases	3233	3087	1611	1575
Total loss of cancer-related life years	5635	5297	2783	2719
Cases of CRC prevented	0	146	1622	1657
Proportion of CRC case prevented (%)	0	4.5	50.2	51.3
Life-years saved	0	339	2852	2917
Number of procedures				
FIT	0	236,913	0	0
M3CRC	0	0	712,584	0
Colonoscopy	0	36,670	419,173	509,496
Diagnostic (without polypectomy)	0	9901	67,487	141,655
Therapeutic (with polypectomy)	0	26,769	351,686	367,841
Number of complications				
Bleeding	0	73	838	1019
Perforations	0	29	335	403
Costs (USD)				
FIT	0	4,247,330	0	0
M3CRC	0	0	37,385,539	0
Colonoscopy	0	40,555,839	408,885,333	526,128,915
Polypectomy	0	3,100,114	35,922,369	39,807,084
Bleeding	0	973,819	9,818,076	12,633,307
Perforations	0	258,360	2,604,796	3,314,825
Care of CRC				
Stage I	4,050,827	3,839,170	2,010,230	1,964,971
Stage II	14,828,981	14,046,379	7,355,408	7,189,266
Stage III	36,148,483	34,219,225	17,920,737	17,514,640
Stage IV	170,486,718	160,919,746	84,332,191	82,394,622
Total	225,515,010	262,159,983	606,234,678	690,947,627
Total costs per life-years saved	-	773,894	212,553	236,909
ICER vs. no screening		108,176	133,485	159,586
ICER vs. FIT			136,896	166,342
ICER vs. M3CRC				1,316,491

CRC: colorectal cancer; FIT: fecal immunochemical tests; ICER: Incremental cost-effectiveness ratio.

## Data Availability

The data that support the findings of this study are available from the corresponding author upon reasonable request.
